# Interesting Cases Successfully Treated with Pepsine

**Published:** 1868-12-15

**Authors:** 


### INTERESTING CASES SUCCESSFULLY TREATED WITH PEPSINE.

SINCE the introduction of *Pepsine* into therapeutics, by Dr. Corvisart, and M. Boudault, chemist, it has been gaining steadily in the estimation of the medical profession. We have transcribed a few very interesting cases, treated with this remedy, from the work of Dr. Corvisart, on "Dyspepsia and Consumption," showing its peculiar adaptability to many disorders of the digestive functions:

CASE I.—Communicated by Dr. A. Longet, member of the Academy of Medicine. *Typhoid Fever.—On the twenty-fourth day, the patient can not yet support any food, even the lightest. The administration of Boudault's Pepsine, in powders, produces immediately easy digestions. On suspending the remedy, as a test, the old symptoms re-appear, with violent pain in the stomach and diarrhœa. The treatment is continued for ten days, when the patient digests perfectly without any help.*—Miss ***, 15 years, pupil of the "Maison Impériale d'Ecouen," on the twenty-fourth day of a serious typhoid affection, although convalescent, was in an alarming state of debility, because she could not support any food, not even the lightest. I ordered her Boudault's *Pepsine* in powders. The first half dose, which was administered in tapioca broth, acted so well, that a second in the same conditions was given to the patient three hours after the first, and was digested without fatigue. The second day the same result, with three broths and a raw egg. The third day the dose was intentionally omitted from the first broth in the morning, and this caused violent pains in the stomach and intestines, and a watery stool. The two others, however, which were administered the same day, and contained each half a dose of *Pepsine*, resulted in a complete and easy digestion. The fourth day of the administration of *Pepsine* the patient ate soups and chicken. After this a more and more substantial food could be given, but every time the dose was suppressed for a meal, the digestion was more or less painful. This state lasted ten days, when the digestion became normal. During this time there existed generally a decided constipation, which, however, gave way under the simplest remedies.

CASE II.—From Dr. Berthelot, of Paris. Miss B. complains of a heaviness in the stomach, and a very difficult digestion, especially of the evening meal. This state, which dated back a whole year, continuing, notwithstanding a varied medication, I prescribed for her one dose of *Pepsine wine* Boudault, at each evening meal. From this time on she digests much better; as soon as she stops these doses, and I have tried it many times, she digests with more difficulty, and the epigastric pains re-appear immediately. The taking of *Pepsine* always renders the digestion painless and easy.

CASE III.—Communicated by Dr. Cahagnet, of Napoléon-Vendée. After a habitual dyspepsia of seven years, with debilitated digestion and loss of strength, disease which resists tonics, purgatives, narcotics, vegetable charcoal, and Seltzer and Vichy Waters, a serious endocarditis sets in. After curing this, the dyspepsia increases; notwithstanding bitters, Vichy Water, etc., no nourishment can be supported; the patient becomes weaker, and vomits every thing, even soups and beef tea. The *Syrup of Pepsine* of Boudault is prescribed for eight days; the digestion is good from the first day, and the alimentation is rendered more copious and more substantial. Strength returns sufficiently to allow walks in the garden.

CASE IV.—From Dr. Parisse, Professor at the "Ecole de Medecine" of Lille. *Obstinate vomiting during pregnancy.*—This case was a young woman of a very weak constitution, of irregular habit, and subjected for a long time to the use of ferruginous preparations, when she became pregnant for the first time. At first I only suspected her state of pregnancy. The stomach troubles become so disquieting, that I prescribed Boudault's *Pepsine* in powders. She used these for fourteen or fifteen days. From the very first day the digestion was better, her condition continued to ameliorate, and soon she could digest without this remedy. It is important to state, that pregnancy had arrived at the fourth month; perhaps the change in the digestion might be attributed to the modifications which the uterus undergoes at this time. Still I do not doubt that the remedy has been really useful.

CASE V.—Communicated by Dr. Huet, adjunct physician to the "Maison Impériale de la Légion d'honneur at Ecouen." Gastralgia of several years standing, resisting the action of antiphlogistic, bitter, ferruginous and antispasmodic remedies: Boudault's *Pepsine* is given, and immediately the digestion is good. The remedy is stopped during four days; all the symptoms re-appear. The *Pepsine* is again taken for twelve days, and the disease disappears. Twenty seven days have elapsed since the remedy has been stopped, and, as no symptoms have re-appeared, the cure may be considered perfect.

CASE VI.—From Dr. O. Landry, ex-interne of the hospitals. Dyspepsia, with painful digestion, swelling, eructations, pain and loss of appetite, and subsequently chlorosis. Calcined magnesia and iron were tried without success. By means of the *Pepsine* Boudault's, the digestion becomes good from the first meal.

CASE VII.—From Dr. Berthelot, of Paris. M. P., thirty-six years of age, of a bilioso-nervous temperament, a great smoker, was for two years troubled with painful digestions, and vomited almost every day after dinner. A lighter diet, and a greater moderation in the use of tobacco were prescribed, but did not in any way help the digestion. I prescribed for him a dose of *Pepsine Boudault*, to be taken at breakfast and at dinner, in the first spoon of soup. During ten days this treatment was continued; the patient digested better, and the vomiting stopped; when the treatment was suspended. Two weeks after he came to thank me; he assured me that he was very well, vomited no more, and digested perfectly.

CASE VIII.—From Dr. A. Godart, corresponding member of the Academy of Medicine. Miss E., vice-principal of a boarding school, thirty years of age, came to consult me the 7th day of January, 1854; she complained of pains in the stomach, which increased but little by pressure. Far from having an appetite; she felt a disgust for all food, the digestion of which was painful, and especially for the evening meal; she slept badly during the night, experienced often spasms and oppression; and could not attend to her duties but with much pain. I prescribed a dose of *Pepsine Boudault* at the beginning of dinner, and morning and evening a pill of extract aconite and stramonium. But the pharmacist, having cautioned the patient to be careful not take more than the prescribed dose, because the remedies were active and dangerous, she was frightened and took none at all. She continued to take the *Pepsine* only for twenty-four days, and to-day, the 23rd day of February, she tells me, that from the second day she felt better, from that time the digestion was re-established, the appetite returned, after some days the spasms and oppression disappeared, she passes comfortable nights, and since the beginning of the month, when she stopped the use of *Pepsine*, she has continued to be pretty well, but the appetite is not as good as when she was still taking it.

